# A Guide to the New Pharmacopœia

**Published:** 1885-12

**Authors:** 


*A Guide to the New Pharmacopœia.* By PROSSER JAMES, M.D., Lecturer on Materia Medica at the London Hospital, &c. London: J. & A. CHURCHILL, New Burlington Street. 1885.

The publication of the new edition of the *British Pharmacopœia* renders it incumbent upon all practitioners and medical students, as well as dispensing chemists, to make themselves acquainted with the differences between the *British Pharmacopœia* of 1885 and its predecessor. These differences are numerous—dispersed throughout the whole work,—and their complete realisation, therefore, a tedious and complicated task. Dr. Prosser James has early come to the rescue of the bewildered investigator, and, Ariadne-like, provides a clue, by which the therapeutical Theseus may be guided through the pharmaceutical labyrinth.

While Dr. P. James has given a synoptical account of the alterations, introductions and omissions which have been made in the new volume, he has not confined himself to mere bald statements, but in most instances has taken occasion, when speaking of a remedy, to give his own experience of its use, and to criticise and comment upon the preparations of it, which may be in the new Pharmacopœia.

We shall, doubtless, soon be having new editions of many of the existing text-books on Materia Medica, revised so as to bring them *en rapport* with the new Pharmacopœia. Pending the publication of such, or to those persons who wish to make the old text-books still continue to serve their purpose, we cordially commend this book. Though published so soon after the other work, upon which it is a commentary, it bears evidence of close study and analysis of subject-matter, and forms a compendious, comprehensive, and critical *vade-mecum* to the alterations in that "inextricabilis error," the *British Pharmacopœia*, 1885.

